# A new estimator for the multicollinear Poisson regression model: simulation and application

**DOI:** 10.1038/s41598-021-82582-w

**Published:** 2021-02-12

**Authors:** Adewale F. Lukman, Emmanuel Adewuyi, Kristofer Månsson, B. M. Golam Kibria

**Affiliations:** 1 Department of Physical Sciences, Landmark University, Omu-Aran, Nigeria; 2 Department of Statistics, Federal University of Technology, Akure, Nigeria; 3 Department of Economics, Finance and Statistics, Jönköping University, Jönköping, Sweden; 4 Department of Mathematics and Statistics, Florida International University, Miami, USA

**Keywords:** Mathematics and computing, Statistics

## Abstract

The maximum likelihood estimator (MLE) suffers from the instability problem in the presence of multicollinearity for a Poisson regression model (PRM). In this study, we propose a new estimator with some biasing parameters to estimate the regression coefficients for the PRM when there is multicollinearity problem. Some simulation experiments are conducted to compare the estimators' performance by using the mean squared error (MSE) criterion. For illustration purposes, aircraft damage data has been analyzed. The simulation results and the real-life application evidenced that the proposed estimator performs better than the rest of the estimators.

## Introduction

The Poisson regression model (PRM) is often adopted in modelling count data. PRM is employed to model the relationship between the response variable and one or more regressors. The response variable comes in the form of a count variable or non-negative integers such as the defects in a unit of manufactured product, errors or bugs in software, number of road accidents, number of times a machine fail in a month, occurrences of virus disease, count of particulate matter or other pollutants in the environment etc. The regression coefficients in PRM are estimated using the Maximum Likelihood Estimator (MLE).

In LRM, the estimator performance suffers from high instability when the regressors are correlated, i.e. multicollinearity (for example, see^1,2^). Multicollinearity effects include significant variance and covariances of the regression coefficients, wider confidence intervals, insignificant *t*-ratios and high R-square. Multicollinearity also negatively influence the performance of the MLE in PRM^3,4^. Alternative estimators to the MLE in LRM are the ridge regression estimator by Hoerl and Kennard^5^, Liu estimator by Liu^6^, Liu type estimator by Liu^7^, two-parameter estimator by Ozkale and Kaciranlar^8^, *k*-*d* class estimator by Sakallioglu and Kaciranlar^9^, a two-parameter estimator by Yang and Chang^10^, modified two-parameter estimator by Dorugade^11^ and recently, the modified ridge type estimator by Lukman et al.^12^, modified new two-parameter estimator by Lukman et al.^13^, modified new two-parameter estimator by Ahmad and Aslam^14^, and K–L estimator by Kibria and Lukman^15^.

Researchers have applied some of these estimators to the Poisson regression model. These include the Poisson ridge regression estimator (PRE) by Månsson and Shukur^3^, Månsson et al.^16^ developed the Poisson Liu estimator (PLE) to mitigate the problem of multicollinearity in PRM. Batah et al.^17^ proposed the modified jackknifed ridge regression estimator (MJRE) for the LRM while Turkan and Özel^18^ adopted the MJRE to the Poisson regression model as a remedy to the problem of multicollinearity. Özkale and Kaciranlar^8^ combine the Liu regression estimator and the ridge regression to form the two-parameter estimator in LRM. Thus, Asar and Genc^19^ implemented the two-parameter estimator to the Poisson regression model. Rashad and Algamal^20^ developed a new ridge estimator for the Poisson regression model by modifying Poisson modified jackknifed ridge regression. Qasim et al.^4^ suggest some new shrinkage estimators for the PLE. We classified these estimators into Poisson regression estimators with a single shrinkage parameter and two-parameters, respectively. Recently, Kibria and Lukman^15^ proposed another ridge type estimator called K–L estimator with a single shrinkage parameter.

This study aims to propose an estimator that can handle multicollinearity in a Poisson regression model. We harmonize the K–L estimator to the PRM and suggest some shrinkage estimators for the estimator. Also, compare the proposed estimator's performance with the MLE, PRE and PLE in terms of the matrix mean square error (MSEM) and mean square error (MSE). The small sample properties are investigated using a simulation experiment. Finally, the new method's benefit is evaluated in an example using aircraft damage data that was initially analyzed by Myers et al.^21^.

This paper structuring is as follows: the Poisson regression model, some estimators and the MSEM and MSE properties of the estimators are discussed in Sect. 2. A Monte Carlo simulation experiment has been conducted in Sect. 3. To illustrate the finding of the paper, aircraft damage data was analyzed in Sect. 4. Some concluding remarks are presented in Sect. 5.

## Statistical methodology

### Poisson regression model and maximum likelihood estimator

Suppose that the response variable, $y_{i}$ is in the form of non-negative integers (or count data), then the probability function is given as follows

2.1 $$f(y_{i} ) = \frac{{\exp \left( { - \mu_{i} } \right)\mu_{i}^{yi} }}{{y_{i} !}},\;y_{i} = 0,1,2, \ldots$$

where $\mu_{i} > 0.$ The mean and variance of the Poisson distribution in Eq. (2.1) are the same (i.e.$E(y) = Var\left( y \right) = \mu$). The model is written in terms of the mean of the response. According to Myers et al.^21^, we assume that there exists a function, *g,* that relates the mean of the response to a linear predictor such that

2.2 $$g\left( {\mu_{i} } \right) = \eta_{i} = \beta_{0} + \beta_{1} x_{1} + \cdots + \beta_{p} x_{p} = x^{\prime}_{i} \beta ,$$

where $g(.)$ is a monotone differentiable link function. The log link function is a popular type of this link function such that $g\left( {\mu_{i} } \right) = \ln \left( {\mu_{i} } \right) = \exp \left( {x^{\prime}_{i} \beta } \right).$ This log link is generally adopted for the Poisson regression model because it ensures that all the fitted values for the response variable are positive. The maximum likelihood estimator is popularly used to estimate the coefficients of the PRM, where the likelihood function is defined as:

2.3 $$l\left( \beta \right) = \prod\limits_{i = 1}^{n} {\frac{{\exp \left( { - \mu_{i} } \right)\mu_{i}^{yi} }}{{y_{i} !}}} . = \frac{{\prod\limits_{i = 1}^{n} {\mu_{i}^{yi} \exp \left( { - \sum\limits_{i = 1}^{n} {\mu_{i} } } \right)} }}{{\prod\limits_{i = 1}^{n} {y_{i} !} }}$$

where $\mu_{i} = g^{ - 1} \left( {x^{\prime}_{i} \beta } \right).$ The log-likelihood function is used to estimate the parameter vector $\beta$

2.4 $$\ln l\left( \beta \right) = \sum\limits_{i = 1}^{n} {y_{i} } \ln \left( {\mu_{i} } \right) - \sum\limits_{i = 1}^{n} {\mu_{i} } - \sum\limits_{i = 1}^{n} {\ln \left( {y_{i} !} \right)}$$

Since Eq. (2.4) is nonlinear in $\beta$, the solution is obtained using iterative methods. A common such procedure is the Fisher Scoring method defined as:

2.5 $$\beta^{t + 1} = \beta^{t} + I^{ - 1} \left( {\beta^{t} } \right)S\left( {\beta^{t} } \right),$$

where $S(\beta ) = \frac{\partial l\left( \beta \right)}{{\partial \beta }}$ and $I^{ - 1} \left( \beta \right) = \left( { - E(\partial^{2} l(\beta )/\partial \beta \partial \beta^{\prime})} \right)^{ - 1}$. The final step of the estimated coefficients corresponds to:

2.6 $$\hat{\beta }^{PMLE} = (X^{\prime}\hat{W}X)^{ - 1} X^{\prime}\hat{W}\hat{z}$$

where $\hat{W} = diag(\mu_{i}^{2} )$ matrix and $\hat{z}$ is the adjusted response variable, $\hat{z} = x^{\prime}_{i} \hat{\beta }^{PMLE} + \frac{{y_{i} - \hat{\mu }_{i} }}{{\hat{\mu }_{i}^{2} }}.$ $\hat{W}$ and $\hat{z}$ are obtained using Fisher scoring iterative procedure (see Hardin and Hilbe^22^). The covariance matrix and mean square error are given respectively as follows:

2.7 $$Cov\left( {\hat{\beta }^{PMLE} } \right) = \left( {X^{\prime}\hat{W}X} \right)^{ - 1}$$

and

2.8 $$MSE\left( {\hat{\beta }^{PMLE} } \right) = \sum\limits_{i = 1}^{p} {\frac{1}{{\lambda_{i} }}}$$

where $\lambda_{i}$ is the ith eigenvalue of the matrix $X^{\prime}\hat{W}X$.

### Poisson K–L estimator

Månsson and Shukur^3^ developed the Poisson ridge regression estimator (PRRE) to mitigate the problem of multicollinearity, which is defined as follows:

2.9 $$\hat{\beta }^{PRRE} = \left( {X^{\prime}\hat{W}X + kI} \right)^{ - 1} X^{\prime}\hat{W}X\hat{\beta }^{PMLE}.$$

where $k > 0$ is the biasing parameter, $I$ is a $p \times p$ identity matrix and the optimal value of *k* is defined as:

2.10 $$k = \frac{1}{{\alpha_{i,\max }^{2} }}$$

where $\hat{\alpha }_{i}$ is the *ith* component of $\alpha = Q^{\prime}\beta$, *Q* is the matrix whose columns are the eigenvectors of $X^{\prime}\hat{W}X.$

Månsson et al.^16^ introduced the Poisson Liu estimator (PLE) as follows:

2.11 $$\hat{\beta }^{PLE} = \left( {X^{\prime}\hat{W}X + I} \right)^{ - 1} \left( {X^{\prime}\hat{W}X + dI} \right)\hat{\beta }^{PMLE} ,\;0 < d < 1,$$

where $d$ according to Månsson et al.^16^ may be estimated by the following formula:

2.12 $$d = \max \left( {0,\min \left( {\frac{{\alpha_{i}^{2} - 1}}{{\frac{1}{{\lambda_{i} }} + \alpha_{i}^{2} }}} \right)} \right)$$

Kibria and Lukman^15^ proposed a new single parameter ridge-type estimator for the linear regression model, which is defined as follows:

2.13 $$\hat{\beta }^{KLE} = (X^{\prime}X + kI_{p} )^{ - 1} (X^{\prime}X - kI_{p} )\hat{\beta }^{MLE}$$

Following Kibria and Lukman^15^, we proposed the following new estimator for the Poisson regression model as follows:

2.14 $$\hat{\beta }^{PKLE} = (X^{\prime}\hat{W}X + kI_{p} )^{ - 1} (X^{\prime}\hat{W}X - kI_{p} )\hat{\beta }^{PMLE}$$

Suppose $\alpha = Q^{\prime}\beta$ and $Q^{\prime}X^{T} \hat{W}XQ = \Lambda = diag\left( {\lambda_{1} ,...,\lambda_{p} } \right)$ where $\lambda_{1} \ge \lambda_{2} \ge ... \ge \lambda_{p} ,\Lambda$ is the matrix of eigenvalues of $X^{T} \hat{W}X$ and *Q* is the matrix whose columns are the eigenvectors of $X^{T} \hat{W}X.$ The matrix mean square error and the mean square error of the estimators PMLE, PRRE, PLE and PKLE are provided in Eqs. (2.15) to (2.21) respectively as follows:

2.15 $$MSEM\left( {\hat{\alpha }^{PMLE} } \right) = Q\Lambda^{ - 1} Q^{T}$$

2.16 $$MSE\left( {\hat{\alpha }^{PMLE} } \right) = \sum\limits_{i = 1}^{p} {\frac{1}{{\lambda_{i} }}}$$

2.17 $$MSEM\left( {\hat{\alpha }^{PRRE} } \right) = Q\Lambda^{k} \Lambda \Lambda^{k} Q^{T} + k^{2} \Lambda^{k} \alpha \alpha^{T} \Lambda^{k}$$

2.18 $$MSE\left( {\hat{\alpha }^{PRRE} } \right) = \sum\limits_{i = 1}^{p} {\left( {\frac{{\lambda_{i} }}{{\left( {\lambda_{i} + k} \right)^{2} }}} \right)} + k^{2} \sum\limits_{i = 1}^{p} {\left( {\frac{{\alpha_{i}^{2} }}{{\left( {\lambda_{i} + k} \right)^{2} }}} \right)}$$

2.19 $$MSEM\left( {\hat{\alpha }^{PLE} } \right) = Q\Lambda_{d} \Lambda^{ - 1} \Lambda_{d}^{T} Q^{T} + \left( {\Lambda_{d} - I} \right)\alpha \alpha^{T} \left( {\Lambda_{d} - I} \right)^{T}$$

where $$\Lambda_{d} = \left( {\Lambda + I} \right)^{ - 1} \left( {\Lambda + dI} \right).$$

2.20 $$MSEM(\hat{\alpha }^{PKLE} ) = Q\Lambda^{k} (\Lambda - kI_{p} )\Lambda^{ - 1} \Lambda^{k} (\Lambda - kI_{p} )Q^{\prime} + 4k^{2} \Lambda^{k} Q\Lambda^{k} \alpha \alpha^{\prime}$$

where $$\Lambda^{k} = \left( {\Lambda + kI_{p} } \right)^{ - 1} .$$

2.21 $$MSE(\hat{\alpha }^{PKLE} ) = \sum\limits_{i = 1}^{p} {\left( {\frac{{\left( {\lambda - k} \right)^{2} }}{{\lambda_{i} \left( {\lambda_{i} + k} \right)^{2} }}} \right)} + 4k^{2} \sum\limits_{i = 1}^{p} {\left( {\frac{{\alpha_{i}^{2} }}{{\left( {\lambda_{i} + k} \right)^{2} }}} \right)}$$

2.22 $$MSE\left( {\hat{\alpha }^{PLE} } \right) = \sum\limits_{j = 1}^{p} {\left( {\frac{{\left( {\lambda_{j} + d} \right)^{2} }}{{\lambda_{j} \left( {\lambda_{j} + 1} \right)^{2} }} + \frac{{\left( {d - 1} \right)^{2} \alpha_{j}^{2} }}{{\left( {\lambda_{j} + 1} \right)^{2} }}} \right)}$$

where $\lambda_{i}$ is the ith eigenvalue of $X^{\prime}\hat{W}X$ and $\alpha_{j}$ is the jth element of $\alpha .$ For the purpose of theoretical comparisons, we adopt the following lemmas.

#### Lemma 2.1

Let *A* be a positive definite (pd) matrix, that is *A* > 0, and $a$ be some vector, then $A - aa^{^{\prime}} \ge 0$ if and only if (iff) $a^{\prime}A^{ - 1} a \le 1$^23^.

#### Lemma 2.2

$MSEM(\hat{\beta }_{1} ) - MSEM(\hat{\beta }_{2} ) = \sigma^{2} D + b_{1} b_{1}^{T} - b_{2} b_{2}^{T} > 0$ if and only if $b_{2}^{T} \left[ {\sigma^{2} D + b_{1} b_{1}^{T} } \right]^{ - 1} b_{2} < 1$ where $MSE\left( {\hat{\beta }_{j} } \right) = Cov\left( {\hat{\beta }_{j} } \right) + b_{j}^{T} b_{j}$^24^.

#### Theorem 2.1

$\hat{\alpha }^{PKLE}$ is better than $\hat{\alpha }^{PMLE}$ iff, $MSEM\;[\hat{\alpha }^{PMLE} ] - MSEM\;[\hat{\alpha }^{PKLE} ] > 0$ provided *k* > 0.

#### Proof.

2.23 $$\begin{gathered} MSEM\left( {\hat{\alpha }^{PMLE} } \right) - MSEM\left( {\hat{\alpha }^{PKLE} } \right) = Q\left[ {\Lambda^{ - 1} - \Lambda^{k} (\Lambda - kI_{p} )\Lambda^{ - 1} \Lambda^{k} (\Lambda - kI_{p} )} \right]Q^{T} \hfill \\ \;\;\;\;\;\;\;\;\;\;\;\;\;\;\;\;\;\;\;\;\;\;\;\;\;\;\;\;\;\;\;\;\;\;\;\;\;\;\;\;\;\;\;\;\;\;\; - 4k^{2} \Lambda^{k} Q\Lambda^{k} \alpha \alpha^{\prime} \hfill \\ \;\;\;\;\;\;\;\;\;\;\;\;\;\;\;\;\;\;\;\;\;\;\;\;\;\;\;\;\;\;\;\;\;\;\;\;\;\;\;\;\;\;\; = Qdiag\left\{ {\frac{1}{{\lambda_{i} }} - \frac{{\left( {\lambda_{i} - k} \right)^{2} }}{{\lambda_{i} \left( {\lambda_{i} + k} \right)^{2} }}} \right\}_{i = 1}^{p} Q^{T} \hfill \\ \;\;\;\;\;\;\;\;\;\;\;\;\;\;\;\;\;\;\;\;\;\;\;\;\;\;\;\;\;\;\;\;\;\;\;\;\;\;\;\;\;\;\;\;\;\; - 4k^{2} \Lambda^{k} Q\Lambda^{k} \alpha \alpha^{\prime} \hfill \\ \end{gathered}$$

The matrix $\Lambda^{ - 1} - \Lambda^{k} (\Lambda - kI_{p} )\Lambda^{ - 1} \Lambda^{k} (\Lambda - kI_{p} )$ is pd since $\lambda_{i} \left( {\lambda_{i} + k} \right)^{2} - \lambda_{i} \left( {\lambda_{i} - k} \right)^{2} > 0.$

#### Theorem 2.2

$\hat{\alpha }^{PKLE}$ is better than $\hat{\alpha }^{PRRE}$ iff, $MSEM\;[\hat{\alpha }^{PRRE} ] - MSEM\;[\hat{\alpha }^{PKLE} ] > 0$ provided *k* > 0.

#### Proof.

2.24 $$\begin{gathered} D\left( {\hat{\alpha }^{PRRE} } \right) - D\left( {\hat{\alpha }^{PKLE} } \right) = Q\left[ {\Lambda^{k} \Lambda \Lambda^{k} - \Lambda^{k} (\Lambda - kI_{p} )\Lambda^{ - 1} \Lambda^{k} (\Lambda - kI_{p} )} \right]Q^{T} \hfill \\ \;\;\;\;\;\;\;\;\;\;\;\;\;\;\;\;\;\;\;\;\;\;\;\;\;\;\;\;\;\;\;\;\;\;\;\;\;\;\;\;\;\;\; = Qdiag\left\{ {\frac{{\lambda_{i} }}{{\left( {\lambda_{i} + k} \right)^{2} }} - \frac{{\left( {\lambda_{i} - k} \right)^{2} }}{{\lambda_{i} \left( {\lambda_{i} + k} \right)^{2} }}} \right\}_{i = 1}^{p} Q^{T} \hfill \\ \end{gathered}$$

The matrix $\Lambda^{k} \Lambda \Lambda^{k} - \Lambda^{k} (\Lambda - kI_{p} )\Lambda^{ - 1} \Lambda^{k} (\Lambda - kI_{p}$ is pd since $\lambda_{i}^{2} \left( {\lambda_{i} + k} \right)^{2} - \left( {\lambda_{i} + k} \right)^{2} \left( {\lambda_{i} - k} \right)^{2} > 0$ for $2\lambda_{i} - k > 0.$

#### Theorem 2.3

$\hat{\alpha }^{PKLE}$ is better than $\hat{\alpha }^{PLE}$ iff, $MSEM\;[\hat{\alpha }^{PLE} ] - MSEM\;[\hat{\alpha }^{PKLE} ] > 0$ provided *k* > 0.

#### Proof.

2.25 $$\begin{gathered} D\left( {\hat{\alpha }^{PLE} } \right) - D\left( {\hat{\alpha }^{PKLE} } \right) = Q\left[ {\Lambda_{d} \Lambda^{ - 1} \Lambda_{d}^{T} - \Lambda^{k} (\Lambda - kI_{p} )\Lambda^{ - 1} \Lambda^{k} (\Lambda - kI_{p} )} \right]Q^{T} \hfill \\ \;\;\;\;\;\;\;\;\;\;\;\;\;\;\;\;\;\;\;\;\;\;\;\;\;\;\;\;\;\;\;\;\;\;\;\;\;\;\;\;\;\;\; = Qdiag\left\{ {\frac{{\left( {\lambda_{j} + d} \right)^{2} }}{{\lambda_{j} \left( {\lambda_{j} + 1} \right)^{2} }} - \frac{{\left( {\lambda_{i} - k} \right)^{2} }}{{\lambda_{i} \left( {\lambda_{i} + k} \right)^{2} }}} \right\}_{i = 1}^{p} Q^{T} \hfill \\ \end{gathered}$$

The matrix found in the above equation $\Lambda_{d} \Lambda^{ - 1} \Lambda_{d}^{T} - \Lambda^{k} (\Lambda - kI_{p} )\Lambda^{ - 1} \Lambda^{k} (\Lambda - kI_{p} )$ is pd since $\lambda_{i} \left( {\lambda_{i} + k} \right)^{2} \left( {\lambda_{j} + d} \right)^{2} - \lambda_{j} \left( {\lambda_{j} + 1} \right)^{2} \left( {\lambda_{i} - k} \right)^{2} > 0.$

### Selection of Biasing Parameter

The parameter is estimated by taking the first derivative of the MSE function of $\hat{\alpha }^{PKLE}$ with respect to *k* and equating the resulting solution to zero. We obtain the following estimates of *k*:

2.26 $$k = \frac{{\lambda_{i} }}{{1 + 2\lambda_{i} \alpha_{i}^{2} }}$$

Following Månsson et al.^16^ and Lukman and Ayinde^25^, we propose the following forms of the shrinkage parameters in Eq. (2.26).

2.27 $$\hat{k}_{1} = \max \left( {0,\min \left( {\frac{{\lambda_{i} }}{{1 + 2\lambda_{i} \alpha_{i}^{2} }}} \right)} \right)$$

2.28 $$\hat{k}_{2} = \sqrt {\max \left( {0,\min \left( {\frac{{\lambda_{i} }}{{1 + 2\lambda_{i} \alpha_{i}^{2} }}} \right)} \right)}$$

## Simulation Experiment

### Simulation Design

Since a theoretical comparison among the estimators is not sufficient, as simulation experiment has been carried out in this section. We generate the response variable of the PRM from the Poisson distribution $P_{0} \left( {\mu_{i} } \right)$ where $\mu_{i} = \exp \left( {x_{i} \beta } \right)\;i = 1,2, \ldots ,n,\;\beta = \left( {\beta_{0} ,\beta_{1} ,\beta_{2} , \ldots ,\beta_{p} } \right)^{\prime }$ such that $x_{i}$ is the ith row of the design matrix *X* and following Kibria^1^, we generated the X matrix as follows:

3.1 $$x_{ij} = \left( {1 - \rho^{2} } \right)^{1/2} w_{ij} + \rho w_{ip + 1} ,\;i = 1,2, \ldots ,n;\;j = 1,2, \ldots p,p + 1$$

where $\rho^{2}$ is the correlation between the explanatory variables. The values of $\rho$ are chosen to be 0.85, 0.9, 0.95 and 0.99. The mean function is obtained for *p* = 4 and 7 regressors, respectively. According to Kibria et al.^26^, the intercept value are chosen to be − 1, 0 and 1 to change the average intensity of the Poisson process. The slope coefficients chosen so that $\sum\nolimits_{j = 1}^{p} {\beta_{j}^{2} = 1}$ and $\beta_{1} = \beta_{2} = \cdots = \beta_{p}$ for sample sizes 50, 75, 100 and 200. Simulation experiment conducted through R programming language^27^. The estimated MSE is calculated as

3.2 $$MSE\left( {\hat{\beta }} \right) = \frac{1}{1000}\sum\limits_{j = 1}^{1000} {\left( {\hat{\beta }_{ij} - \beta_{i} } \right)^{\prime } } \left( {\hat{\beta }_{ij} - \beta_{i} } \right)$$

where $\hat{\beta }_{ij}$ denotes the estimate of the ith parameter in jth replication and β_i_ is the true parameter values. The experiment is replicated 1000 times. The simulated MSE values of the estimators for p = 4 and intercepts = − 1, 0 and 1 are presented in Tables 1, 2, 3 respectively and p = 7 and intercepts = − 1, 0 and 1 are presented in Tables 4, 5, 6, respectively.

**Table 1:** Simulated MSE when p = 4 and intercept = − 1.

Intercept	n	ρ	PKLE1	PKLE2	PLE	PRRE	PMLE
− 1	50	0.8	0.2688	0.2324	0.2668	0.2691	0.3194
		0.9	0.3422	0.2780	0.3468	0.3445	0.4434
		0.95	0.4729	0.3391	0.4902	0.4812	0.6854
		0.99	1.5356	0.5282	1.5721	1.5470	2.8210
		0.999	15.5580	2.9798	15.6323	15.3346	28.6901
	75	0.8	0.1772	0.1635	0.1805	0.1777	0.2134
		0.9	0.2477	0.2237	0.2528	0.2493	0.3067
		0.95	0.3547	0.3028	0.3640	0.3593	0.4681
		0.99	0.9971	0.5388	1.0494	1.0258	1.7516
		0.999	8.3318	0.6540	8.3224	8.2732	15.8245
	100	0.8	0.1644	0.1520	0.1623	0.1644	0.1763
		0.9	0.2273	0.2076	0.2274	0.2278	0.2571
		0.95	0.3323	0.2894	0.3366	0.3345	0.4074
		0.99	1.0515	0.5912	1.1219	1.0801	1.7301
		0.999	7.8180	0.7432	7.9173	7.7334	15.0254
	200	0.8	0.0429	0.0420	0.0423	0.0429	0.0435
		0.9	0.0535	0.0527	0.0535	0.0535	0.0557
		0.95	0.0816	0.0800	0.0827	0.0817	0.0879
		0.99	0.2728	0.2438	0.2763	0.2749	0.3274
		0.999	1.7187	0.6548	1.7933	1.7500	3.1157

**Table 2:** Simulated MSE when p = 4 and intercept = 0.

Intercept	n	ρ	PKLE1	PKLE2	PLE	PRRE	PMLE
0	50	0.8	0.0701	0.0683	0.0707	0.0702	0.0756
		0.9	0.1003	0.0955	0.1012	0.1007	0.1138
		0.95	0.1715	0.1561	0.1741	0.1735	0.2143
		0.99	0.5111	0.3241	0.5317	0.5315	0.9181
		0.999	4.6909	0.6275	4.6140	4.6207	9.0882
	75	0.8	0.0546	0.0537	0.0547	0.0546	0.0570
		0.9	0.0801	0.0780	0.0803	0.0802	0.0856
		0.95	0.1303	0.1245	0.1308	0.1307	0.1456
		0.99	0.3850	0.3168	0.3976	0.3972	0.5741
		0.999	3.0237	0.6477	2.9929	3.0076	5.6671
	100	0.8	0.0418	0.0413	0.0419	0.0418	0.0431
		0.9	0.0690	0.0675	0.0691	0.0691	0.0727
		0.95	0.1168	0.1122	0.1174	0.1171	0.1290
		0.99	0.3912	0.3238	0.4055	0.4034	0.5806
		0.999	2.8662	0.6173	2.8339	2.8511	5.4321
	200	0.8	0.0102	0.0102	0.0102	0.0102	0.0103
		0.9	0.0147	0.0146	0.0147	0.0147	0.0149
		0.95	0.0265	0.0263	0.0265	0.0265	0.0270
		0.99	0.1015	0.0978	0.1017	0.1017	0.1108
		0.999	0.6370	0.4352	0.6620	0.6618	1.1017

**Table 3:** Simulated MSE when p = 4 and intercept = 1.

Intercept	n	ρ	PKLE1	PKLE2	PLE	PRRE	PMLE
1	50	0.8	0.0411	0.0408	0.0411	0.0411	0.0416
		0.9	0.0519	0.0511	0.0519	0.0519	0.0532
		0.95	0.0822	0.0793	0.0823	0.0822	0.0868
		0.99	0.2734	0.2298	0.2818	0.2784	0.3567
		0.999	1.7816	0.4751	1.8210	1.7884	3.4578
	75	0.8	0.0269	0.0268	0.0269	0.0269	0.0271
		0.9	0.0390	0.0387	0.0391	0.0390	0.0396
		0.95	0.0596	0.0586	0.0596	0.0596	0.0613
		0.99	0.2104	0.1944	0.2118	0.2113	0.2389
		0.999	1.2893	0.6147	1.3499	1.3232	2.3019
	100	0.8	0.0235	0.0234	0.0235	0.0235	0.0236
		0.9	0.0343	0.0340	0.0343	0.0343	0.0345
		0.95	0.0519	0.0512	0.0519	0.0519	0.0528
		0.99	0.2060	0.1900	0.2073	0.2066	0.2279
		0.999	1.1375	0.5447	1.2443	1.1820	2.0549
	200	0.8	0.0057	0.0056	0.0057	0.0057	0.0057
		0.9	0.0076	0.0076	0.0076	0.0076	0.0076
		0.95	0.0115	0.0115	0.0115	0.0115	0.0116
		0.99	0.0421	0.0415	0.0421	0.0421	0.0430
		0.999	0.3123	0.2704	0.3185	0.3159	0.3868

**Table 4:** Simulated MSE when p = 7 and intercept = − 1.

Intercept	n	ρ	PKLE1	PKLE2	PLE	PRRE	PMLE
− 1	50	0.8	0.5026	0.4263	0.4988	0.5018	0.6064
		0.9	0.7669	0.5998	0.7795	0.7688	1.0486
		0.95	1.2641	0.8189	1.2873	1.2721	1.9715
		0.99	6.3135	1.5820	6.1834	6.2140	10.7269
		0.999	64.7209	21.1207	64.3927	63.0823	112.6646
	75	0.8	0.2380	0.2162	0.2388	0.2385	0.2770
		0.9	0.3137	0.2732	0.3264	0.3177	0.4234
		0.95	0.4385	0.3349	0.4646	0.4486	0.7010
		0.99	2.1826	0.8014	2.1642	2.1503	3.9059
		0.999	23.5750	9.5019	22.9197	22.6216	42.8199
	100	0.8	0.1463	0.1413	0.1478	0.1464	0.1610
		0.9	0.2107	0.2019	0.2149	0.2113	0.2386
		0.95	0.3366	0.3134	0.3505	0.3396	0.4186
		0.99	1.2396	0.8224	1.2956	1.2697	1.9434
		0.999	12.1942	1.9688	11.9290	11.9912	20.8725
	200	0.8	0.0516	0.0506	0.0512	0.0516	0.0524
		0.9	0.0757	0.0744	0.0759	0.0757	0.0791
		0.95	0.1279	0.1240	0.1285	0.1281	0.1362
		0.99	0.4777	0.4019	0.4916	0.4866	0.6330
		0.999	4.0605	1.3613	3.9903	3.9785	6.9296

**Table 5:** Simulated MSE when p = 7 and intercept = 0.

Intercept	n	ρ	PKLE1	PKLE2	PLE	PRRE	PMLE
0	50	0.8	0.1489	0.1447	0.1503	0.1492	0.1617
		0.9	0.2452	0.2323	0.2483	0.2467	0.2835
		0.95	0.4212	0.3767	0.4310	0.4288	0.5537
		0.99	1.8687	0.9555	1.8648	1.8765	3.2182
		0.999	20.4742	3.3127	19.9500	20.0905	35.4611
	75	0.8	0.0658	0.0644	0.0661	0.0659	0.0699
		0.9	0.1094	0.1044	0.1103	0.1099	0.1236
		0.95	0.1797	0.1623	0.1833	0.1827	0.2302
		0.99	0.7702	0.4389	0.7854	0.7866	1.3652
		0.999	8.0166	2.3384	7.5503	7.6873	14.7306
	100	0.8	0.0489	0.0484	0.0489	0.0489	0.0501
		0.9	0.0755	0.0744	0.0755	0.0755	0.0782
		0.95	0.1306	0.1272	0.1308	0.1307	0.1394
		0.99	0.5228	0.4570	0.5342	0.5335	0.7070
		0.999	4.4932	1.4207	4.3748	4.4380	7.5910
	200	0.8	0.0134	0.0133	0.0134	0.0134	0.0135
		0.9	0.0227	0.0226	0.0227	0.0227	0.0230
		0.95	0.0401	0.0397	0.0401	0.0401	0.0412
		0.99	0.1930	0.1819	0.1945	0.1943	0.2241
		0.999	1.4410	0.7989	1.4362	1.4478	2.4561

**Table 6:** Simulated MSE when p = 7 and intercept = 1.

Intercept	n	ρ	PKLE1	PKLE2	PLE	PRRE	PMLE
1	50	0.8	0.0792	0.0784	0.0792	0.0792	0.0805
		0.9	0.1247	0.1218	0.1248	0.1248	0.1296
		0.95	0.2244	0.2126	0.2253	0.2249	0.2448
		0.99	0.8773	0.6405	0.9176	0.9017	1.3239
		0.999	8.8188	1.8730	8.4501	8.6064	15.0688
	75	0.8	0.0347	0.0344	0.0347	0.0347	0.0351
		0.9	0.0518	0.0508	0.0518	0.0518	0.0534
		0.95	0.0983	0.0941	0.0985	0.0984	0.1055
		0.99	0.3866	0.2998	0.4050	0.3981	0.5553
		0.999	3.2853	1.0512	3.1833	3.1984	5.9012
	100	0.8	0.0218	0.0217	0.0218	0.0218	0.0218
		0.9	0.0322	0.0321	0.0322	0.0322	0.0325
		0.95	0.0547	0.0541	0.0547	0.0547	0.0556
		0.99	0.2388	0.2251	0.2398	0.2393	0.2612
		0.999	1.7042	0.9834	1.7764	1.7417	2.7838
	200	0.8	0.0072	0.0072	0.0072	0.0072	0.0072
		0.9	0.0103	0.0103	0.0103	0.0103	0.0104
		0.95	0.0171	0.0170	0.0171	0.0171	0.0172
		0.99	0.0814	0.0793	0.0814	0.0814	0.0846
		0.999	0.6326	0.4903	0.6622	0.6495	0.9012

### Simulation results discussion

**Figure 1:**
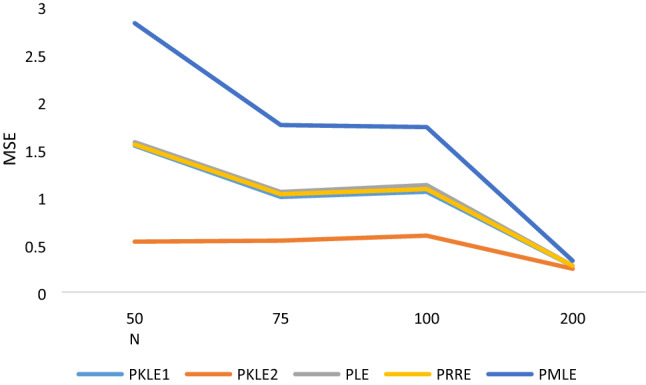
Intercept = − 1; ρ = 0.95; p = 4.

**Figure 2:**
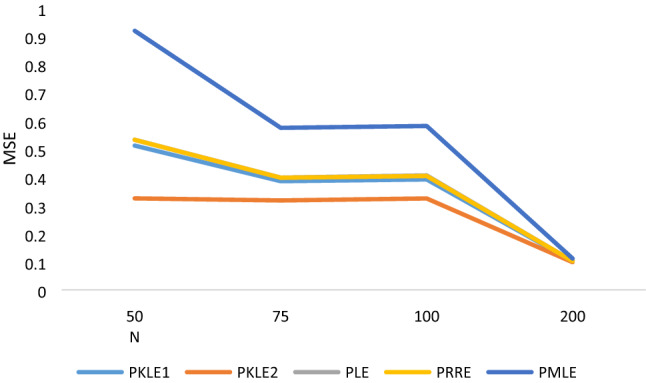
Intercept = 0; ρ = 0.99; p = 4.

**Figure 3:**
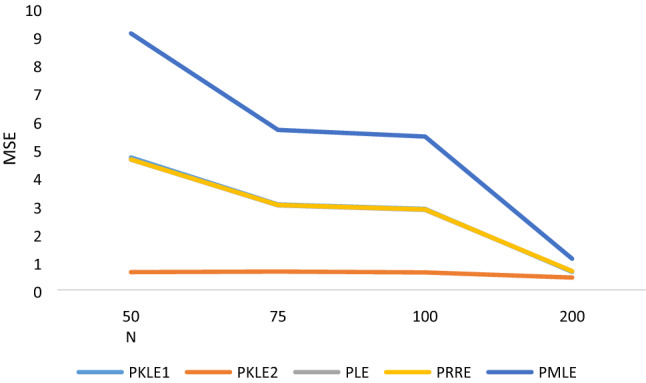
Intercept = 0; ρ = 0.999; p = 7.

**Figure 4:**
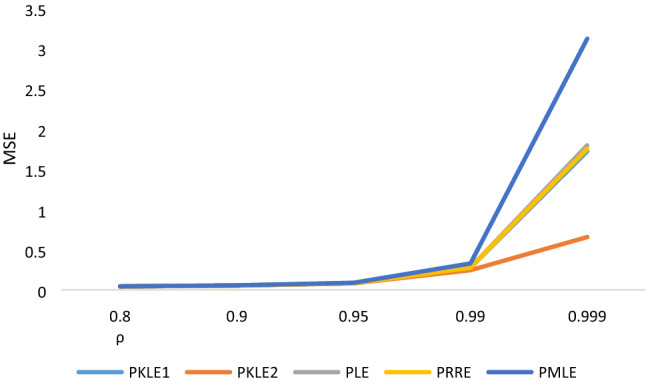
Intercept = 1; n = 200; p = 4.

**Figure 5:**
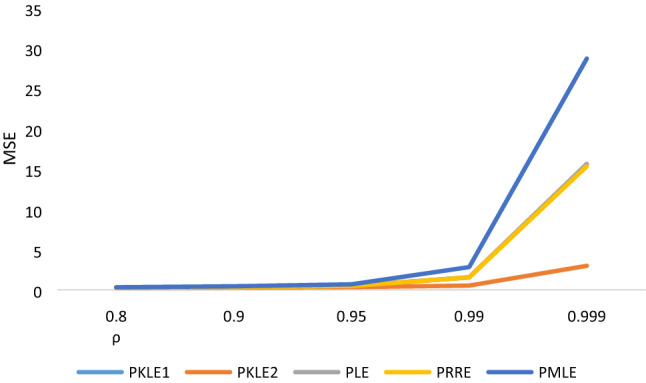
Intercept = 1; n = 200; p = 7.

The simulation result in Tables 1, 2, 3, 4, 5, 6 shows that the following factors affect the estimators’ performances: the degree of correlation, the number of explanatory variables, the sample size and the value of the intercept. We observed that increasing the sample size led to a decrease in the MSE values of all the estimators, which is one of the unique properties for any statistical estimator. The proposed estimator, PKLE2 consistently possessed the minimum MSE. Increasing the degree of correlation increases the simulated MSE values for each of the estimators. The Poisson ridge (PRE) and Liu estimator (PLE) competes favorably with the proposed estimator. For instance, The MSE of PRE and PLE are very similar to the proposed estimator, especially when multicollinearity is low (ρ = 0.8–0.95).The performance of PMLE is the worst compared to other estimators, especially when the correlation among regressors is 0.90 and higher. This study increased explanatory variables from 4 to 7 and observed that the MSE rises by increasing explanatory variables. The MSE for all the estimators decreases when we change the intercept value from − 1 to + 1. Consistently, the proposed estimator PKLE2 outperforms all other estimators considered in this study. We also plotted MSE vs sample sizes and different ρ and intercepts and presented them Figs. 1, 2, 3, 4 and 5. From these figures, we observed that PKLE2 consistently possessed minimum value at the different sample size (*n*), followed by PKLE1 while PMLE has the worst performance. These figures also revealed that the estimators’ performance becomes similar for large *n* (200) or small correlation (0.80). However, the proposed estimator, PKLE2 performed the best.

## Real life application

In this session, we examined the effectiveness of the new estimator using real-life data. We adopted the aircraft damage data to evaluate the proposed estimator's performance and some other estimators in this study. The dataset was initially used by Myers et al.^21^ and recently by Asar and Genc^19^ and others. The dataset provides the information about two types of aircraft, the McDonnell Douglas A-4 Skyhawk and the Grumman A-6 Intruder. This data describe 30 strike missions of these two aircraft. The explanatory variables are as follows: *x*_1_ is a binary variable representing the aircraft type (A-4 coded as 0 and A-6 coded as 1), *x*_2_ and *x*_3_ denote bomb load in tons and total months of aircrew experience, respectively. The response variable, *y* represents the number of locations with damage on the aircraft, which follows a Poisson distribution^19,21^. Amin et al.^28^ examine if the model follows a Poisson regression model by adopting the Pearson chi-square goodness of fit test. The test confirms that the response variable is well fitted to the Poisson distribution with test statistic (p-value) is given as 6.89812 (0.07521).

According to Myers et al.^21^, there is evident of multicollinearity problem in the data. The eigenvalues of the $X^{\prime}\hat{W}X$ matrix are 4.3333, 374.8961 and 2085.2251. The condition number, $CN = \sqrt {\frac{{\max \left( {eigenvalue} \right)}}{{\min \left( {eigenvalue} \right)}}} = 219.365$, also shows multicollinearity in the dataset^2,12^. The estimators’ performances are assessed through the mean squared error (MSE). The MSE of the estimators is computed using Eqs. (2.15). (2.17), (2.19) and (2.21), respectively. The biasing parameters are determined using Eqs. (2.10), (2.12), (2.27) and (2.28), respectively. The regression coefficients and the MSE values are provided in Table 7. From Table 7, we observed that all the coefficients have a similar sign. PMLE has the highest mean square error, while the proposed estimator (PKLE2) has the lowest MSE which established its superiority. The maximum likelihood estimator possesses the highest MSE due to the presence of multicollinearity. The ridge and Liu estimator equally perform well when there is multicollinearity. We observed that the performance of the proposed estimator is a function of the biasing parameter, *k*.

**Table 7:** Regression coefficients and MSE.

Coef.	$\hat{\alpha }^{PMLE}$	$\hat{\alpha }^{PRRE}$	$\hat{\alpha }^{PLE}$	$\hat{\alpha }^{PKLE1}$	$\hat{\alpha }^{PKLE2}$
$\hat{\alpha }_{0}$	− 0.4060	− 0.1676	− 0.2555	− 0.1085	− 0.1068
$\hat{\alpha }_{1}$	0.5688	0.3799	0.4789	0.3921	0.3906
$\hat{\alpha }_{2}$	0.1654	0.1705	0.1665	0.1675	0.1675
$\hat{\alpha }_{3}$	− 0.0135	− 0.0153	− 0.0147	− 0.0158	− 0.0158
MSE	1.0290	0.2727	0.4320	0.2251	0.2249

## Some concluding remarks

The K–L estimator is an estimator with a single biasing parameter, *k *which eliminates the biasing parameter's computational rigour as obtainable in some of the two-parameter estimators. It falls in the ridge and Liu estimator class to mitigate multicollinearity in the linear regression model. According to Kibria and Lukman^15^, K–L estimator outclasses the following estimators: the ordinary least squares estimator, the ridge and the Liu estimator in the linear regression model. As earlier stated, the multicollinearity influences the performance of the maximum likelihood estimator (MLE) in both the linear regression models and the Poisson regression models (PRM). The ridge regression and Liu estimator at a different time were harmonized to the PRM to solve multicollinearity. However, in this study, we developed a new estimator, establish its statistical properties, carried out theoretical comparisons with the estimators mentioned above. Furthermore, we conducted a simulation experiment and analyzed a real-life application to show the proposed estimator effectiveness. The simulated and application results show that the proposed estimators outperform the existing estimators, while PMLE has the worst performance.

## References

[1] Kibria BMG (2003). Performance of some new ridge regression estimators. Commun. Stat. Simul. Comput..

[2] Lukman AF, Ayinde K, Aladeitan BB, Rasak B (2020). An unbiased estimator with prior information. Arab J. Basic Appl. Sci..

[3] Månsson K, Shukur G (2011). A Poisson ridge regression estimator. Econ. Model..

[4] Qasim M, Kibria BMG, Månsson K, Sjölander P (2019). A new Poisson Liu regression estimator: method and application. J. Appl. Stat..

[5] Hoerl AE, Kennard RW (1970). Ridge regression: biased estimation for nonorthogonal problems. Technometrics.

[6] Liu K (1993). A new class of biased estimate in linear regression. Commun. Stat..

[7] Liu K (2003). Using Liu-type estimator to combat collinearity. Commun. Stat..

[8] Ozkale MR, Kaciranlar S (2007). The restricted and unrestricted two-parameter estimators. Commun. Statist. Theor. Meth..

[9] Sakallıoğlu S, Kaçıranlar S (2008). A new biased estimator based on ridge estimation. Statist. Papers.

[10] Yang H, Chang X (2010). A new two-parameter estimator in linear regression. Commun. Stat. Theory Methods.

[11] Dorugade AV (2014). Modified two parameter estimator in linear regression. J. Stat. Trans. New Ser..

[12] Lukman AF, Ayinde K, Binuomote S, Onate AC (2019). Modified ridge-type estimator to combat multicollinearity: application to chemical data. J. Chemomet..

[13] Lukman AF, Ayinde K, Sek SK, Adewuyi E (2019). A modified new two-parameter estimator in a linear regression model. Model. Simul. Eng..

[14] Ahmad S, Aslam M (2020). Another proposal about the new two-parameter estimator for linear regression model with correlated regressors. Commun. Stat. Simul. Comput..

[15] Kibria BMG, Lukman AF (2020). A new ridge-type estimator for the linear regression model: simulations and applications. Scientifica.

[16] Månsson, Kibria, B.M.G., Sjölander, P. and Shukur, G. (2012). Improved Liu estimators for the Poisson regression model. *Int. J. Stat. Prob.* 1(1).

[17] Batah FSM, Ramanathan TV, Gore SD (2008). The efficiency of modified Jackknife and ridge type regression estimators: a comparison. Surv. Math. Appl..

[18] Türkan S, Özel G (2016). A new modified Jackknifed estimator for the Poisson regression model. J. Appl. Stat..

[19] Asar Y, Genc A (2017). A new two-parameter estimator for the poisson regression model. Iran J Sci Technol Trans Sci.

[20] Rashad NK, Algamal ZY (2019). A new ridge estimator for the poisson regression model. Iran J. Sci. Technol. Trans. Sci..

[21] Myers RH, Montgomery DC, Vining GG, Robinson TJ (2012). Generalized linear models: With applications in engineering and the sciences, 791.

[22] Hardin JW, Hilbe JM (2012). Generalized linear models and extensions.

[23] Farebrother RW (1976). Further results on the mean square error of ridge regression. J. Roy. Statist. Soc. B.

[24] Trenkler G, Toutenburg H (1990). Mean squared error matrix comparisons between biased estimators—an overview of recent results. Stat Pap..

[25] Lukman AF, Ayinde K (2017). Review and classifications of the ridge parameter estimation techniques. Hacettepe J. Math. Stat..

[26] Kibria BMG, Månsson K, Shukur G (2015). A simulation study of some biasing parameters for the ridge type estimation of Poisson regression. Commun. Stat. Simul Comput. I.

[27] R Core Team (2020). R: A Language and Environment for Statistical Computing. R Foundation for Statistical Computing, Vienna, Austria. https://www.R-project.org.

[28] Amin M, Akram MN, Amanullah M (2020). On the James-Stein estimator for the Poisson regression model. Commun. Stat. Simul. Comput..

